# Using cascading Bloom filters to improve the memory usage for de Brujin graphs

**DOI:** 10.1186/1748-7188-9-2

**Published:** 2014-02-24

**Authors:** Kamil Salikhov, Gustavo Sacomoto, Gregory Kucherov

**Affiliations:** 1 Lomonosov Moscow State University, Moscow, Russia; 2 INRIA Grenoble Rhône-Alpes, Grenoble, France; 3 Laboratoire Biométrie et Biologie Evolutive, Université Lyon 1, Lyon, France; 4 Department of Computer Science, Ben-Gurion University of the Negev, Be’er Sheva, Israel; 5 Laboratoire d’Informatique Gaspard Monge, Université Paris-Est & CNRS, Marne-la-Vallée, Paris, France

**Keywords:** Next-generation sequencing, Genome assembly, de Brujin graph, Bloom filter

## Abstract

**Background:**

De Brujin graphs are widely used in bioinformatics for processing next-generation sequencing data. Due to a very large size of NGS datasets, it is essential to represent de Bruijn graphs compactly, and several approaches to this problem have been proposed recently.

**Results:**

In this work, we show how to reduce the memory required by the data structure of Chikhi and Rizk (WABI’12) that represents de Brujin graphs using Bloom filters. Our method requires 30% to 40% less memory with respect to their method, with insignificant impact on construction time. At the same time, our experiments showed a better query time compared to the method of Chikhi and Rizk.

**Conclusion:**

The proposed data structure constitutes, to our knowledge, currently the most efficient practical representation of de Bruijn graphs.

## Background

Modern next-generation sequencing (NGS) technologies generate huge volumes of short nucleotide sequences (*reads*) drawn from a DNA sample under study. The length of a read varies from 35 to about 400 base pairs (letters) and the number of reads may be hundreds of millions, thus the total volume of data may reach tens or even hundreds of Gb.

Many computational tools dealing with NGS data, especially those devoted to *genome assembly*, are based on the concept of a *de Bruijn graph*, see e.g. [1]. Nodes of a de Bruijn graph^a^ correspond to all distinct *k-mers* occurring in the given set of reads, and two *k-mers* are linked by an arc if they have a suffix-prefix overlap of size *k*−1. The value of *k* is an open parameter that in practice is chosen between 20 and 64. The idea of using de Bruijn graphs for genome assembly goes back to the “pre-NGS era” [2]. Note, however, that *de novo* genome assembly is not the only application of those graphs when dealing with NGS data. There are several others, including: *de novo* transcriptome assembly [3] and *de novo* alternative splicing calling [4] from transcriptomic NGS data (RNA-seq); metagenome assembly [5] from metagenomic NGS data; and genomic variant detection [6] from genomic NGS data using a reference genome.

Due to a very large size of NGS datasets, it is essential to represent de Bruijn graphs as compactly as possible. This has been a very active line of research. Recently, several papers have been published that propose different approaches to compressing de Bruijn graphs [7-11].

Conway and Bromage [7] proposed a method based on classical succinct data structures, i.e. bitmaps with efficient rank/select operations. On the same direction, Bowe et al. [10] proposed an interesting succinct representation that, assuming only one string (read) is present, uses only 4*E* bits, where *E* is the number of arcs in the graph. The more realistic case where there are *R* reads can be easily reduced to the one string case by concatenating all *R* reads using a special separator character. In this case, however, the size of the structure is 4*E*+*O*(*R* log*E*) bits ([10], Theorem 1). Since the multiplicative constant of the second term is difficult to evaluate, it is hard to know precisely what would be the size of this structure in practice.

Ye et al. [8] proposed a different method based on a sparse representation of de Bruijn graphs, where only a subset of *k*-mers present in the dataset are stored. Using the *Bloom filter* data structure, Pell et al. [11] proposed *probabilistic de Bruijn graphs* as a compact approximate representation of full de Bruijn graphs. Finally, Chikhi and Rizk [9] improved Pell’s scheme in order to obtain an exact representation of the de Bruijn graph.

A direct application of Bloom filters to de Bruijn graphs, studied in [11], results in a space-efficient representation at the price of allowing *one-sided errors*, namely *false positive* nodes (*k*-mers). The method of [9] removes these errors and proposes a space-efficient data structure for the *exact* representation of de Bruijn graphs. The method is based on the following idea. In the genome assembly application, de Bruijn graphs are only used for traversal, and random accesses to graph nodes are never performed. If all queried nodes (*k*-mers) are only those which are reachable from some node known to belong to the graph, then only a fraction of all false positives can actually occur. Storing these false positives explicitly leads to an exact (false positive free) and space-efficient representation of the de Bruijn graph. This is the best practical *exact* representation of de Bruijn graphs for the purpose of genome assembly, now implemented in MINIA software [15].

Our main contribution is an improvement of the method of [9] by changing the representation of the set of false positives. We achieve this by iteratively applying a Bloom filter to represent the set of false positives, then the set of “false false positives” etc. We show analytically that this cascade of Bloom filters allows for a considerable further economy of memory, improving the method of [9]. Depending on the value of *k*, our method requires 30% to 40% less memory with respect to the method of [9]. Moreover, with our method, the memory grows very little as *k* grows. Finally, we implemented our method and tested it against [9] on real datasets. The tests confirm the theoretical predictions for the size of structure and show a 20% to 30% *improvement* in query times.

## Preliminaries

A *Bloom filter* is a space-efficient data structure for representing a given subset of elements *T*⊆*U*, with support for efficient membership queries with one-sided error. That is, if a query for an element *x*∈*U* returns *no* then *x*∉*T*, but if it returns *yes* then either *x*∈*T*, or, with small probability, *x*∉*T* (false positive). A Bloom filter consists of a bitmap (array of bits) *B* of size *m* and a set of *p* distinct hash functions {*h*_1_,…,*h*_*p*_}, where *h*_*i*_:*U*↦{0,…,*m*−1}. Initially, all bits of *B* are set to 0. An insertion of an element *x*∈*T* is done by setting the bits of *B* with indices *h*_1_(*x*),…,*h*_*p*_(*x*) to 1, i.e. *B*[ *h*_*i*_(*x*)]=1 for all *i*∈ [ 1,*p*]. Membership queries are done symmetrically, returning *yes* if all *B*[ *h*_*i*_(*x*)] are equal 1 and *no* otherwise. As shown in [12], when considering hash functions that yield equally likely positions in the bitmap, and for large enough bitmap size *m* and number of inserted elements *n*, the false positive rate  is

(1) $$F\approx {\left(1-e^{-\text{pn}/m}\right)}^{p}={\left(1-e^{-p/r}\right)}^{p}$$

where *r*=*m*/*n* is the number of bits (of the bitmap *B*) per element (of *T* represented). It is not hard to see that this expression is minimized when *p*=*r* ln2, giving the false positive rate

(2) $$F\approx {\left(1-e^{-\ln2}\right)}^{r\ln2}={\left(1/2\right)}^{r\ln2}\approx 0.6185^{r}.$$

A *de Bruijn graph*, for a given parameter *k*, of a set of reads (strings) $R\subseteq \Sigma^{*}={\left\{A,C,T,G\right\}}^{*}$ is entirely defined by the set *T*⊆*U*=*Σ*^*k*^ of *k*-mers present in . The nodes of the graph are precisely the *k*-mers of *T* and for any two vertices *u*,*v*∈*T*, there is an arc from *u* to *v* if the suffix of *u* of size (*k*−1) is equal to the prefix of *v* of the same size. Thus, given a set *T*⊆*U* of *k*-mers, we can represent its de Bruijn graph using a Bloom filter *B*. This approach has the disadvantage of having false positive nodes, as a direct consequence of false positives in the Bloom filter, which can create false connections in the graph (see [11] for the influence of false positive nodes on the topology of the graph). The naive way to remove those false positives nodes by explicitly storing (e.g. using a hash table) the set of all false positives of *B* is clearly inefficient, as the expected number of elements to be explicitly stored is $|U|F=4^{k}F$.

The key idea of [9] is to explicitly store only a small subset of all false positives of *B*, the so-called *critical false positives*. Consider a *k*-mer *u* that belongs to *T*, *u* has at most 2|*Σ*|=8*potential neighbors*, i.e. *k*-mers overlapping *u* by (*k*−1) letters. The set of *critical false positives* consists of the potential neighbors of *k*-mers of *T* that are false positives of *B*. This set is, in general, much smaller than the set of all false positives of *B*, its expected size can be upper-bounded by 8|T|F. On the other hand, storing the set of critical false positives is clearly sufficient to represent the de Bruijn graph if one only wants to support graph traversal, i.e. navigation from a node of the graph to its neighbors. In this case, only potential neighbors of nodes in *T* are queried.

## Cascading Bloom filter

Let  be a set of reads and *T*_0_ be the set of occurring *k*-mers (nodes of the de Brujin graph) that we want to store. As stated in Section “Preliminaries”, the method of [9] stores *T*_0_ via a bitmap *B*_1_ using a Bloom filter, together with the set *T*_1_ of critical false positives. *T*_1_ consists of potential neighbors of *T*_0_ which are stored in *B*_1_ “by mistake”, i.e. belong^b^ to *B*_1_ but not to *T*_0_. *B*_1_ and *T*_1_ are sufficient to represent the graph provided that the only queried *k*-mers are those which are potential neighbors of *k*-mers of *T*_0_.

The idea we introduce in this work is to use this structure recursively and represent the set *T*_1_ by a new bitmap *B*_2_ and a new set *T*_2_, then represent *T*_2_ by *B*_3_ and *T*_3_, and so on. More formally, starting from *B*_1_ and *T*_1_ defined as above, we define a series of bitmaps *B*_1_,*B*_2_,… and a series of sets *T*_1_,*T*_2_,… as follows. *B*_2_ stores the set of false positives *T*_1_ using another Bloom filter, and *T*_2_ contains the critical false positives of *B*_2_, i.e. true positives from *T*_0_ that are stored in *B*_2_ “by mistake” (we call them *false false positives*). *B*_3_ and *T*_3_, and, generally, *B*_*i*_ and *T*_*i*_ are defined similarly: *B*_*i*_ stores *k*-mers of *T*_*i*−1_ using a Bloom filter, and *T*_*i*_ contains *k*-mers stored in *B*_*i*_ “by mistake”, i.e. those *k*-mers in *B*_*i*_ that do not belong to *T*_*i*−1_ but belong to *T*_*i*−2_. Observe that *T*_0_∩*T*_1_=*∅*, *T*_0_⊇*T*_2_⊇*T*_4_… and *T*_1_⊇*T*_3_⊇*T*_5_….

The following lemma shows that the construction is correct, that is it allows one to verify whether or not a given *k*-mer belongs to the set *T*_0_.

### **Lemma 1**.

Given a *k*-mer (node) *u*, consider the smallest *i* such that *u*∉*B*_*i*+1_ (if *u*∉*B*_1_, we define *i*=0). Then, if *i* is odd, then *u*∈*T*_0_, and if *i* is even (including 0), then *u*∉*T*_0_.

### *Proof*.

Observe that *u*∉*B*_*i*+1_ implies *u*∉*T*_*i*_ by the basic property of Bloom filters that membership queries have one-sided error, i.e. there are no false negatives. We first check the Lemma for *i*=0,1.

For *i*=0, we have *u*∉*B*_1_, and then *u*∉*T*_0_.

For *i*=1, we have *u*∈*B*_1_ but *u*∉*B*_2_. The latter implies that *u*∉*T*_1_, and then *u* must be a false false positive, that is *u*∈*T*_0_. Note that here we use the fact that the only queried *k*-mers *u* are either nodes of *T*_0_ or their potential neighbors in the graph (see [9]), and therefore if *u*∈*B*_1_ and *u*∉*T*_0_ then *u*∈*T*_1_.

For the general case *i*≥2, we show by induction that *u*∈*T*_*i*−1_. Indeed, *u*∈*B*_1_∩…∩*B*_*i*_ implies *u*∈*T*_*i*−1_∪*T*_*i*_ (which, again, is easily seen by induction), and *u*∉*B*_*i*+1_ implies *u*∉*T*_*i*_.

Since *T*_*i*−1_⊆*T*_0_ for odd *i*, and *T*_*i*−1_⊆*T*_1_ for even *i* (for *T*_0_∩*T*_1_=*∅*), the lemma follows.

Naturally, Lemma 1 provides an algorithm to check if a given *k*-mer *u* belongs to the graph: it suffices to check successively if it belongs to *B*_1_,*B*_2_,… until we encounter the first *B*_*i*+1_ which does not contain *u*. Then, the answer will simply depend on whether *i* is even or odd: *u* belongs to the graph if and only if *i* is odd

In our reasoning so far, we assumed an infinite number of bitmaps *B*_*i*_. Of course, in practice we cannot store infinitely many (and even simply many) bitmaps. Therefore, we truncate the construction at some step *t* and store a finite set of bitmaps *B*_1_,*B*_2_,…,*B*_*t*_ together with an explicit representation of *T*_*t*_. The procedure of Lemma 1 is extended in the obvious way: if for all 1≤*i*≤*t*, *u*∈*B*_*i*_, then the answer is determined by directly checking *u*∈*T*_*t*_.

## Analysis of the data structure

### Memory and time usage

First, we estimate the memory needed by our data structure, under the assumption of an infinite number of bitmaps. Let *N* be the number of true positives, i.e. |*T*_0_|=*N*. As stated in Section “Preliminaries”, if *T*_0_ has to be stored via a bitmap *B*_1_ of size *rN*, the false positive rate can be estimated as *c*^*r*^, where *c*=0.6185. And, the expected number of critical false positive nodes (set *T*_1_) has been estimated in [9] to be 8*Nc*^*r*^, as every node has eight extensions, i.e. potential neighbors in the graph. We slightly refine this estimation to 6*Nc*^*r*^ by noticing that for most of the graph nodes, two out of these eight extensions belong to *T*_0_ (are real nodes) and thus only six are potential false positives. Furthermore, to store these 6*Nc*^*r*^ critical false positive nodes, we use a bitmap *B*_2_ of size 6*rNc*^*r*^. Bitmap *B*_3_ is used for storing nodes of *T*_0_ which are stored in *B*_2_ “by mistake” (set *T*_2_). We estimate the number of these nodes as the fraction *c*^*r*^ (false positive rate of filter *B*_2_) of *N* (size of *T*_0_), that is *Nc*^*r*^. Similarly, the number of nodes we need to put to *B*_4_ is 6*Nc*^*r*^ multiplied by *c*^*r*^, i.e. 6*Nc*^2*r*^. Keeping counting in this way, the memory needed for the whole structure is *rN*+6*rNc*^*r*^+*rNc*^*r*^+6*rNc*^2*r*^+*rNc*^2*r*^+… bits. The number of bits per *k*-mer is then

(3) $$\begin{array}{c} r\;+\;6rc^{r}\;+\;rc^{r}\;+\;6rc^{2r}+\ldots =(r+6rc^{r})(1\;+\;c^{r}+c^{2r}+\ldots )\\ \;=(1+6c^{r})\frac{r}{1-c^{r}}. \end{array}$$

A simple calculation shows that the minimum of this expression is achieved when *r*=5.464, and then the minimum memory used per *k*-mer is 8.45 bits.

As mentioned earlier, in practice we store only a finite number of bitmaps *B*_1_,…,*B*_*t*_ together with an explicit representation (such as array or hash table) of *T*_*t*_. In this case, the memory taken by the bitmaps is a truncated sum *rN*+6*rNc*^*r*^+*rNc*^*r*^+.., and a data structure storing *T*_*t*_ takes either $2k\cdot Nc^{\lceil \frac{t}{2}\rceil r}$ or $2k\cdot 6Nc^{\lceil \frac{t}{2}\rceil r}$ bits, depending on whether *t* is even or odd. The latter follows from the observations that we need to store $Nc^{\lceil \frac{t}{2}\rceil r}$ (or $6Nc^{\lceil \frac{t}{2}\rceil r}$) *k*-mers, each taking 2*k* bits of memory. Consequently, we have to adjust the optimal value of *r* minimizing the total space, and re-estimate the resulting space spent on one *k*-mer.

Table 1 shows estimations for optimal values of *r* and the corresponding space per *k*-mer for *t*=4 and *t*=6, and several values of *k*. The data demonstrates that even such small values of *t* lead to considerable memory savings. It appears that the space per *k*-mer is very close to the optimal space (8.45 bits) obtained for the infinite number of filters. Table 1 reveals another advantage of our improvement: the number of bits per stored *k*-mer remains almost constant for different values of *k*.

**Table 1:** **1st column: *k *-mer size; 2nd and 4th columns: optimal value of *r * for Bloom filters (bitmap size per number of stored elements) for *t *** **=4 and *t *** **=6 respectively; 3rd and 5th columns: the resulting space per *k *-mer (for *t *** **=4 and *t *** **=6); 6th column: space per *k *-mer for the method of [**[9]**] ( *t *** **=1)**

***k***	**Optimal** ***r***	**Bits per *k *-mer**	**Optimal** ***r***	**Bits per *k *-mer**	**Bits per *k *-mer**
	**for *t *** **=4**	**for *t *** **=4**	**for *t *** **=6**	**for *t *** **=6**	**for *t *** **=1 (**[9]**)**
16	5.777	8.556	5.506	8.459	12.078
32	6.049	8.664	5.556	8.470	13.518
64	6.399	8.824	5.641	8.490	14.958
128	6.819	9.045	5.772	8.524	16.398

The last column of Table 1 shows the memory usage of the original method of [9], obtained using the estimation $\left(1.44\underset{2}{\log}\left(\frac{16k}{2.08}\right)+2.08\right)$ the authors provided. Note that according to that estimation, doubling the value of *k* results in a memory increment by 1.44 bits, whereas in our method the increment is of 0.11 to 0.22 bits.

Let us now comment on query and preprocessing times for our scheme. The query time can be split into two parts: the time spent on querying *t* Bloom filters and the time spent on querying *T*_*t*_. As stated in Section “Preliminaries”, each query in a Bloom filter corresponds to *p*=*r* ln2 hash functions evaluations. Clearly, the total query time for *t* Bloom filters is *tp*=*Θ*(*tr*). Thus, it is expected that using *t* Bloom filters, even if *r* decreases, the query time increases. For instance, with *t*=4 we have that *r*=6.049 (*k*=32) and the total number of hash function evaluations is proportional to *rt*≈24, whereas with *t*=1 we have that *r*=11.44 and *rt*≈12, a factor 2 increase in the number of hash function evaluations. On the other hand, the set *T*_*t*_ is generally much smaller than *T*_1_, due to the above-mentioned exponential decrease. Depending on the data structure for storing *T*_*t*_, the time saving in querying *T*_*t*_ vs. *T*_1_ may even dominate the time loss in querying multiple Bloom filters. Our experimental results (Section “Construction algorithm” below) confirm that this situation does indeed occur in practice. Note that even in the case when querying *T*_*t*_ weakly depends on its size (e.g. when *T*_*t*_ is implemented by a hash table), the query time will not increase much, due to our choice of a small value for *t*, as discussed earlier.

At the preprocessing step, we need to construct Bloom filters *B*_1_,…,*B*_*t*_ and set *T*_*t*_. At each stage *i*, we need to store *T*_*i*−1_ and *T*_*i*−2_ (possibly on disk, if we want to save on the internal memory used by the algorithm) to construct *B*_*i*_ and *T*_*i*_. A key observation is that the sizes of *B*_*i*_ and *T*_*i*_ decrease exponentially on *i* and therefore the time spent to construct the whole structure is a linear function on the size of *T*_0_. In particular, asymptotically it is only a small constant factor larger compared to the original method of [9]. If the value of *t* is small (such as *t*=4, as in Table 1), the preprocessing time is obviously even smaller.

### Using different values of *r* for different filters

In the previous section, we assumed that each of our Bloom filters uses the same value of *r*, the ratio of bitmap size to the number of stored *k*-mers. However, formula (3) for the number of bits per *k*-mer shows a difference for odd and even filter indices. This suggests that using different parameters *r* for different filters, rather than the same for all filters, may reduce the space even further. If *r*_*i*_ denotes the corresponding ratio for filter *B*_*i*_, then (3) should be rewritten to

(4) $$r_{1}+6r_{2}c^{r_{1}}+r_{3}c^{r_{2}}+6r_{4}c^{r_{1}+r_{3}}+\ldots ,$$

and the minimum value of this expression becomes 7.93 (this value is achieved with *r*_1_=4.41;*r*_*i*_=1.44,*i*>1).

In the same way, we can use different values of *r*_*i*_ in the truncated case. This leads to a small 2% to 4% improvement in comparison with the case of unique value of *r*. Table 2 shows results for the case *t*=4 for different values of *k*.

**Table 2:** **Estimated memory occupation for the case of different values of *r * vs. single value of *r * (shown in Table 1), for 4 Bloom filters ( *t *** **=4)**

***k***	**Optimal** ***r***_**1**_**,** ***r***_**2**_**,** ***r***_**3**_**,** ***r***_**4**_	**Bits per *k *-mer**	**Bits per *k *-mer**
		**different values of** ***r***	**single value of** ***r***
16	5.254, 3.541, 4.981, 8.653	8.336	8.556
32	5.383, 3.899, 5.318, 9.108	8.404	8.664
64	5.572, 4.452, 5.681, 9.108	8.512	8.824
128	5.786, 5.108, 6.109, 9.109	8.669	9.045

### Query distribution among filters

The query algorithm of Lemma 1 simply queries Bloom filters *B*_1_,…,*B*_*t*_ successively as long as the returned answer is positive. The query time then directly depends on the number of filters applied before getting a negative answer. Therefore, it is instructive to analyse how the query frequencies to different filters are distributed when performing a graph traversal. We provide such an analysis in this section.

We analyse query frequencies during an exhaustive traversal of the de Bruijn graph, when each true node is visited exactly once. We assume that each time a true node is visited, all its eight potential neighbors are queried, as there is no other way to tell which of those neighbors are real. Note however that this assumption does not take into account structural properties of the de Bruijn graph, nor any additional statistical properties of the genome (such as genomic word frequencies).

For a filter *B*_*i*_, we want to estimate the number of queried *k*-mers resolved by *B*_*i*_ during the traversal, that is queries on which *B*_*i*_ returns *no*. This number is the difference of the number of queries submitted to *B*_*i*_ and the number of queries for which *B*_*i*_ returns *yes*. Note that the queries submitted to *B*_*i*_ are precisely those on which the previous filter *B*_*i*−1_ returns *yes*.

If the input set *T*_0_ contains *Nk*-mers, then the number of queries in a graph traversal is 8*N*, since for each true node each of its 8 potential neighbors are queried. Moreover, about 2*N* queries correspond to true *k*-mers, as we assume that most of the graph nodes have two true neighbors. Filter *B*_1_ will return *yes* on 2*N*+6*c*^*r*^*N* queries, corresponding to the number of true and false positives respectively. For an arbitrary *i*, filter *B*_*i*_ returns *yes* precisely on the *k*-mers inserted to *B*_*i*_ (i.e. *k*-mers *B*_*i*_ is built on), and the *k*-mers which are inserted to *B*_*i*+1_ (which are the critical false positives for *B*_*i*_). The counts then easily follow from the analysis of Section “Memory and time usage”.

Table 3 provides counts for the first four filters, together with the estimated fraction of *k*-mers resolved by each filter (last row), for the case of infinite number of filters. The data shows that 99.48*%* of all *k*-mers are resolved by four filters, which suggests that a very small number of filters is sufficient to cover a vast majority of *k*-mers. Furthermore, Table 4 shows data for 1-, 2- and 4-filter setups, this time with the optimal value of *r* for each case. Even two filters are already sufficient to reduce the accesses to *T*_2_ to 2.08*%*. In case of four filters, 99.7*%* of *k*-mers are resolved before accessing *T*_4_.

**Table 3:** **Estimations of the number of queries made to filters** ***B***_**1**_**,** ***B***_**2**_**,** ***B***_**3**_**,** ***B***_**4**_** and of the fraction of queries resolved by each filter (for the optimal value *r *** **=5 *. *** **464), in the case of infinite number of filters**

	***B***_**1**_	***B***_**2**_	***B***_**3**_	***B***_**4**_
nb of queries	8*N*	(2+6*c*^*r*^)*N*	(6*c*^*r*^+2*c*^*r*^)*N*	(2*c*^*r*^+6*c*^2*r*^)*N*
Queries returning *yes*	(2+6*c*^*r*^)*N*	(6*c*^*r*^+2*c*^*r*^)*N*	(2*c*^*r*^+6*c*^2*r*^)*N*	(6*c*^2*r*^+2*c*^2*r*^)*N*
Queries returning *no*	(6−6*c*^*r*^)*N*	(2−2*c*^*r*^)*N*	(6*c*^*r*^−6*c*^2*r*^)*N*	(2*c*^*r*^−2*c*^2*r*^)*N*
Fraction of resolved queries	69.57*%*	23.19*%*	5.04*%*	1.68*%*

**Table 4:** **Estimated fractions of queries resolved by each filter and by the explicitely stored set** ***T***_***t***_** for *t *** **=1,2,4, computed for *k *** **=32 and optimal value of *r * shown in the second column**

**Value of** ***t***	***r***	***B***_**1**_	***B***_**2**_	***B***_**3**_	***B***_**4**_	***T***_***t***_
1	11.44	74.70*%*	0	0	0	25.3*%*
2	8.060	73.44*%*	24.48*%*	0	0	2.08*%*
4	6.049	70.90*%*	23.63*%*	3.88*%*	1.29*%*	0.3*%*

## Experimental results

### Construction algorithm

In practice, constructing a cascading Bloom filter for a real-life read set is a computationally intensive step. To perform it on a commonly-used computer, the implementation makes an essential use of external memory. Here we give a short description of the construction algorithm for up to four Bloom filters. Extension for larger number of filters is straightforward.

We start from the input set *T*_0_ of *k*-mers written on disk. In this set, for each pair of *k*-mer and its reverse complement we keep only one of them, the lexicographically smaller, and identify the other to it. We build the Bloom filter *B*_1_ of appropriate size by inserting elements of *T*_0_ successively. Next, all possible extensions of each *k*-mer in *T*_0_ are queried against *B*_1_, and those which return true are written to the disk. Then, in this set only the *k*-mers absent from *T*_0_ are kept, i.e. we perform a set difference from *T*_0_. We cannot afford to load *T*_0_ entirely in memory, so we partition *T*_0_ and perform the set difference in several iterations, loading only one partition of *T*_0_ each time. This results in the set *T*_1_ of critical false positives, which is also kept on disk. Up to this point, the procedure is identical to that of [9].

Next, we insert all *k*-mers from *T*_1_ into *B*_2_ and to obtain *T*_2_, we check for each *k*-mer in *T*_0_ if a query to *B*_2_ returns true. This results in the set *T*_2_, which is directly stored on disk. Thus, at this point we have *B*_1_, *B*_2_ and, by loading *T*_2_ from the disk, a complete representation for *t*=2. In order to build the data structure for *t*=4, we continue this process, by inserting *T*_2_ in *B*_3_ and retrieving (and writing directly on disk) *T*_3_ from *T*_1_ (stored on disk). It should be noted that to obtain *T*_*i*_ we need *T*_*i*−2_, and by always directly storing it on disk we guarantee not to use more memory than the size of the final structure. The set *T*_*t*_ (that is, *T*_1_, *T*_2_ or *T*_4_ in our experiments) is represented as a sorted array and is searched by a binary search. We found this implementation more efficient than a hash table.

### Implementation and experimental setup

We implemented our method using MINIA software [9] and ran comparative tests for 2 and 4 Bloom filters (*t*=2,4). Note that since the only modified part of MINIA was the construction step and the *k*-mer membership queries, this allows us to precisely evaluate our method against the one of [9].

The first step of the implementation is to retrieve the list of *k*-mers that appear more than *d* times using DSK [13] – a constant memory streaming algorithm to count *k*-mers. Note, as a side remark, that performing counting allows us to perform off-line deletions of *k*-mers. That is, if at some point of the scan of the input set of *k*-mers (or reads) some of them should be deleted, it is done by a simple decrement of the counter.

Assessing the query time is done through the procedure of graph traversal, as it is implemented in [9]. Since the procedure is identical and independent on the data structure, the time spent on graph traversal is a faithful estimator of the query time.

We compare three versions: *t*=1 (i.e. the version of [9]), *t*=2 and *t*=4. For convenience, we define 1 Bloom, 2 Bloom and 4 Bloom as the versions with *t*=1,2 and 4, respectively.

### *E.coli* dataset, varying *k*

**Figure 1:**
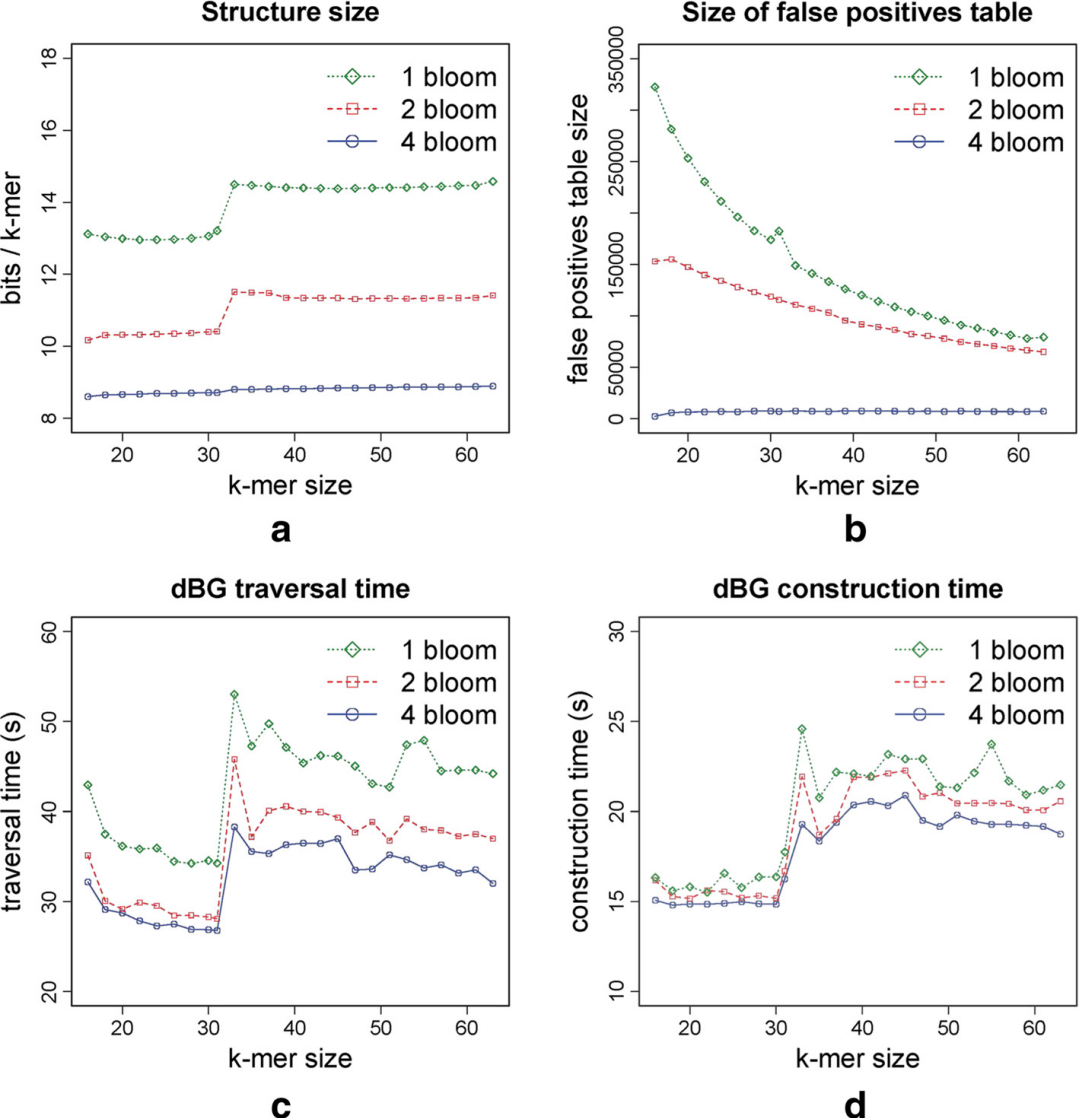
**Results for 10M E.coli reads of 100bp using several values of*k*.** The *1 Bloom* version corresponds to the one presented in [9]. **(a)** Size of the structure in bits used per *k*-mer stored. **(b)** Number of false positives stored in *T*_1_, *T*_2_ or *T*_4_ for 1, 2 or 4 Bloom filters, respectively. **(c)** De Bruijn graph traversal time, including branching *k*-mer indexing. **(d)** De Bruijn graph construction time, excluding *k*-mer counting step.

In this set of tests, our main goal was to evaluate the influence of the *k*-mer size on principal parameters: size of the whole data structure, size of the set *T*_*t*_, graph traversal time, and time of construction of the data structure. We retrieved 10M *E. coli* reads of 100bp from the *Short Read Archive* (ERX008638) without read pairing information and extracted all *k*-mers occurring at least two times. The total number of *k*-mers considered varied, depending on the value of *k*, from 6,967,781 (*k*=15) to 5,923,501 (*k*=63). We ran each version, 1 Bloom [9], 2 Bloom and 4 Bloom, for values of *k* ranging from 16 to 64. The results are shown in Figure 1.

The total size of the structures in bits per stored *k*-mer, i.e. the size of *B*_1_ and *T*_1_ (respectively, *B*_1_,*B*_2_, *T*_2_ or *B*_1_,*B*_2_,*B*_3_,*B*_4_, *T*_4_) is shown in Figure 1(a). As expected, the space for 4 Bloom filters is the smallest for all values of *k* considered, showing a considerable improvement, ranging from 32% to 39%, over the version of [9]. Even the version with just 2 Bloom filters shows an improvement of at least 20% over [9], for all values of *k*. Regarding the influence of the *k*-mer size on the structure size, we observe that for 4 Bloom filters the structure size is almost constant, the minimum value is 8.60 and the largest is 8.89, an increase of only 3%. For 1 and 2 Bloom the same pattern is seen: a plateau from *k*=16 to 32, a jump for *k*=33 and another plateau from *k*=33 to 64. The jump at *k*=32 is due to switching from 64-bit to 128-bit representation of *k*-mers in the table *T*_*t*_.

Figure 1(b) shows the size of table *T*_*t*_ (number of *k*-mers) for *t*=1,2,4, depending on *k*. It clearly demonstrates the sharp decrease of the size of *T*_*t*_ with growing *t*, in accordance with the exponential decrease estimated analytically in Section “Memory and time usage”. We also observe a decrease in the size of *T*_*t*_ with growing *k* for *t*=1 and, to a smaller extent, for *t*=2, while for *t*=4 the decrease is not noticeable. This is explained by the increase rate of optimal *r* (Table 1) which is high for *t*=1, smaller for *t*=2 and yet smaller for *t*=4. Since the size of *T*_*t*_ is *O*(*Nc*^*tr*/2^) (Section “Memory and time usage”) for *c*<1 and almost invariable *N*, the decrease rate is exponential w.r.t. the increase rate of *r*.

Traversal times for each version are shown in Figure 1(c). The fastest version is 4 Bloom, showing an improvement over [9] of 18% to 30%, followed by 2 Bloom. This result is surprising and may seem counter-intuitive, as we have four filters to apply to the queried *k*-mer rather than a single filter as in [9]. However, the size of *T*_4_ (or even *T*_2_) is much smaller than *T*_1_, as the size of *T*_*i*_’s decreases exponentially. As *T*_*t*_ is stored in an array, the time economy in searching *T*_4_ (or *T*_2_) compared to *T*_1_ dominates the time lost on querying additional Bloom filters, which explains the overall gain in query time.

As far as the construction time is concerned (Figure 1(d)), our versions yielded also a faster construction, with the 4 Bloom version being 5% to 22% faster than that of [9]. The gain is explained by the time required for sorting the array storing *T*_*t*_, which is much higher for *T*_0_ than for *T*_2_ or *T*_4_. However, the gain is less significant here, and, on the other hand, was not observed for bigger datasets (see Section “Human dataset”).

### *E. coli* dataset, varying coverage

**Figure 2:**
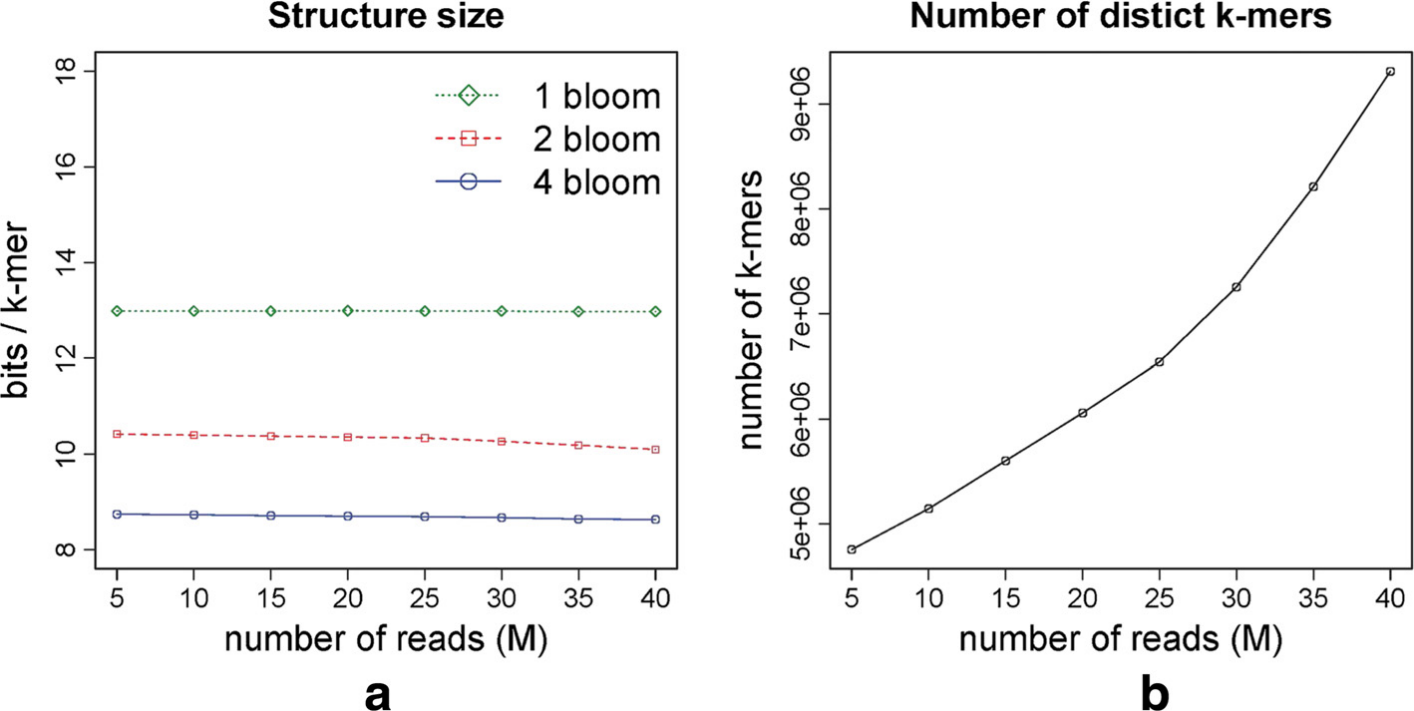
**Results for*E.coli* reads of 100bp using*k*=27.** The *1 Bloom* version corresponds to the one presented in [9]. **(a)** Size of the structure in bits used per *k*-mer stored. **(b)** Number of distinct *k*-mers.

From the complete *E. coli* dataset (≈44M reads) from the previous section, we selected several samples ranging from 5M to 40M reads in order to assess the impact of the coverage on the size of the data structures. This strain *E. coli* (K-12 MG1655) is estimated to have a genome of 4.6M bp [14], implying that a sample of 5M reads (of 100bp) corresponds to ≈100X coverage. We set *d*=3 and *k*=27. The results are shown in Figure 2. As expected, the memory consumption per *k*-mer remains almost constant for increasing coverage, with a slight decrease for 2 and 4 Bloom. The best results are obtained with the 4 Bloom version, an improvement of 33% over the 1 Bloom version of [9]. On the other hand, the number of distinct *k*-mers increases markedly (around 10% for each 5M reads) with increasing coverage, see Figure 2(b). This is due to sequencing errors: an increase in coverage implies more errors with higher coverage, which are not removed by our cutoff *d*=3. This suggests that the value of *d* should be chosen according to the coverage of the sample. Moreover, in the case where read qualities are available, a quality control pre-processing step may help to reduce the number of sequencing errors.

### *E. coli* dataset, query statistics

**Figure 3:**
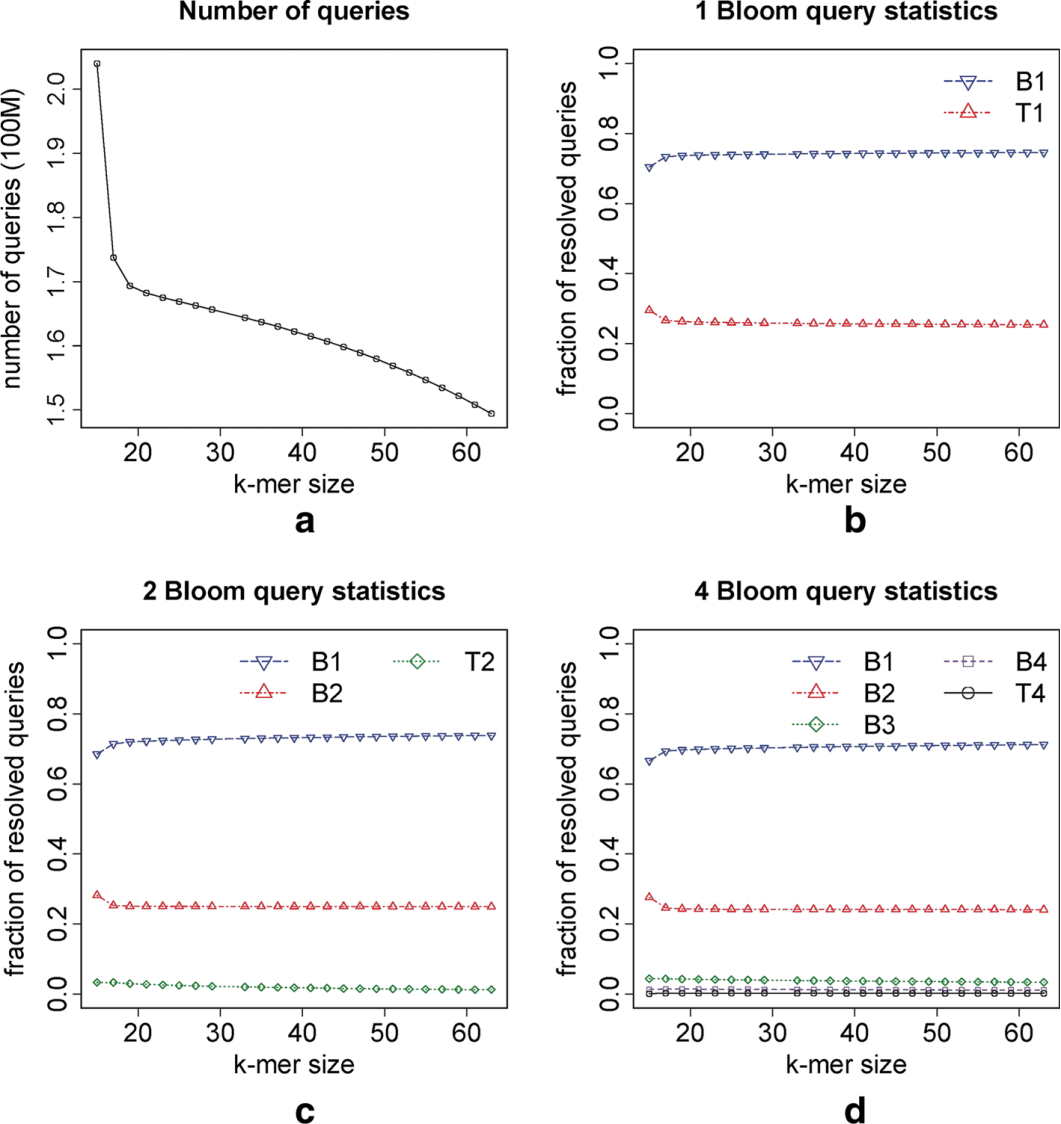
**Query statistics results for 10M E.coli reads of 100bp using several values of*k*.** The *1 Bloom* version corresponds to the one presented in [9]. **(a)** Total number of queries performed, for each value of *k*, during a graph traversal. **(b)** Fraction of resolved queries in *B*_1_ and *T*_1_ (1 Bloom version) for each value of *k*. **(c)** Fraction of resolved queries in *B*_1_, *B*_2_ and *T*_2_ (2 Bloom version) for each value of *k*. **(d)** Fraction of resolved queries in *B*_1_, *B*_2_, *B*_3_, *B*_4_ and *T*_4_ for each value of *k*.

In this set of tests we used the dataset of Section “*E.coli* dataset, varying *k*” to experimentally evaluate how the queries are distributed among the Bloom filters. We ran the graph traversal algorithm for each version, 1 Bloom [9], 2 Bloom and 4 Bloom, using values of *k* ranging from 16 to 64 and retrieved the number of queries resolved in each Bloom filter and the table *T*_*t*_. The results are shown in Figure 3. The plots indicate that, for each version, the query distribution among the Bloom filters is approximately invariant to the value of *k*. Indeed, on average 74%, 73% and 70% of the queries are resolved in *B*_1_ for the 1, 2 and 4 Bloom version, respectively, and the variance is smaller than 0.01% in each case. For the 4 Bloom version, 70%, 24%, 4%, 1% and 0.2% of the queries are resolved in *B*_1_, *B*_2_, *B*_3_, *B*_4_ and *T*_4_, respectively, showing that the values estimated theoretically in Section “Query distribution among filters” (the last row of Table 4) are very precise. Furthermore, as a query to a Bloom filter is faster than to *T*_1_ and the majority of the queries to 4 and 2 Bloom versions, 94% and 95% respectively, are resolved in the first two filters, it is natural that on average queries to 1 Bloom version are slower than to 2 and 4 Bloom versions, corroborating the results of Section “*E.coli* dataset, varying *k*”.

### Human dataset

We also compared 2 and 4 Bloom versions with the 1 Bloom version of [9] on a large dataset. For that, we retrieved 564M Human reads of 100bp (SRA: SRX016231) without pairing information and discarded the reads occurring less than 3 times. The dataset corresponds to ≈17X coverage. A total of 2,455,753,508 *k*-mers were indexed. We ran each version, 1 Bloom [9], 2 Bloom and 4 Bloom with *k* = 23. The results are shown in Table 5.

**Table 5:** **Results of 1, 2 and 4 Bloom filters version for 564M Human reads of 100 bp using *k *** **=23**

**Method**	**1 Bloom **[9]	**2 Bloom**	**4 Bloom**
Construction time (s)	40160.7	43362.8	44300.7
Traversal time (s)	46596.5	35909.3	34177.2
*r* coefficient	11.10	7.80	5.97
	*B*_1_=3250.95	*B*_1_=2283.64	*B*_1_=1749.04
		*B*_2_=323.08	*B*_2_=591.57
Bloom filters size (MB)			*B*_3_=100.56
			*B*_4_=34.01
False positive table	*T*_1_=545.94	*T*_2_=425.74	*T*_4_=36.62
size (MB)			
Total size (MB)	3796.89	3032.46	2511.8
**Size (bits/ *k *-mer)**	**12.96**	**10.35**	**8.58**

The results are in general consistent with the previous tests on *E.coli* datasets. There is an improvement of 34% (21%) for the 4 Bloom (2 Bloom) in the size of the structure. The graph traversal is also 26% faster in the 4 Bloom version. However, in contrast to the previous results, the graph construction time increased by 10% and 7% for 4 and 2 Bloom versions respectively, when compared to the 1 Bloom version. This is due to the fact that disk writing/reading operations now dominate the time for the graph construction, and 2 and 4 Bloom versions generate more disk accesses than 1 Bloom. As stated in Section “Construction algorithm”, when constructing the 1 Bloom structure, the only part written on the disk is *T*_1_ and it is read only once to fill an array in memory. For 4 Bloom, *T*_1_ and *T*_2_ are written to the disk, and *T*_0_ and *T*_1_ are read at least one time each to build *B*_2_ and *B*_3_. Moreover, since the size coefficient of *B*_1_ reduces, from *r*=11.10 in 1 Bloom to *r*=5.97 in 4 Bloom, the number of false positives in *T*_1_ increases.

## Discussion and conclusions

Using cascading Bloom filters for storing de Bruijn graphs has clear advantages over the single-filter method of [9]. In terms of memory consumption, which is the main parameter here, we obtained an improvement of around 30%-40% in all our experiments. Our data structure takes 8.5 to 9 bits per stored *k*-mer, compared to 13 to 15 bits by the method of [9]. This confirms our analytical estimations. The above results were obtained using only four filters and are very close to the estimated optimum (around 8.4 bits/*k*-mer) produced by the infinite number of filters. This is consistent with both our analytical estimations and experimental data showing that over 99% of queries are resolved by the four filters, without resorting to the explicitely stored set *T*_*t*_. Even two filters only resolve about 95% of queries. An interesting characteristic of our method is that the memory grows insignificantly with the growth of *k*, even slower than with the method of [9]. Somewhat surprisingly, we also obtained a significant decrease, of order 20%-30%, of query time. The construction time of the data structure varied from being 10% slower (for the human dataset) to 22% faster (for the bacterial dataset). Cascading Bloom filters have now been implemented by default in the MINIA software [15].

As stated previously, another compact encoding of de Bruijn graphs has been proposed in [10], however no implementation of the method was made available. For this reason, we could not experimentally compare our method with the one of [10]. We remark, however, that the space bound of [10] heavily depends on the number of reads (i.e. coverage), while in our case, the data structure size is almost invariant with respect to the coverage (Section “*E. coli* dataset, varying coverage”).

An interesting open question is whether the Bloom filter construction can be made online, so that new *k*-mers (reads) can be inserted without reconstructing the whole data structure from scratch. Note that the presented construction (Section “Construction algorithm”) is inherently off-line, as all *k*-mers should be known before the data structure is built.

Another interesting prospect for possible further improvements of our method is offered by work [16], where an efficient replacement to Bloom filter was introduced. The results of [16] suggest that we could hope to reduce the memory to about 5 bits per *k*-mer. However, there exist obstacles on this way: an implementation of such a structure would probably result in a significant construction and query time increase.

## Endnotes

^a^ Note that this is actually a *subgraph* of the de Bruijn graph under its classical combinatorial definition. However, we still call it de Bruijn graph to follow the terminology common to the bioinformatics literature.

^b^ By a slight abuse of notation, we also view *B*_*j*_ as the set of all *k*-mers on which the filter *B*_*j*_ returns the positive answer.

## Competing interests

The authors declare that they have no competing interests.

## Authors’ contributions

KS and GK designed the data structure. KS performed analytical estimatons. GS implemented the method and performed the computational experiments with biological data. All authors contributed to writing the manuscript. All authors read and approved the final manuscript.
